# Fabry Disease Podocytes Reveal Ferroptosis as a Potential Regulator of Cell Pathology

**DOI:** 10.1016/j.ekir.2024.11.024

**Published:** 2024-11-23

**Authors:** Andrea F. Wise, IGAA Ari Krisnadevi, Shoni Bruell, Han-Chung Lee, Tejasvini Bhuvan, Andrew J. Kassianos, Sheetal Saini, Xiangju Wang, Helen G. Healy, Elizabeth Ling Qian, David A. Elliot, Joel R Steele, Maria Fuller, Kathleen M. Nicholls, Sharon D. Ricardo

**Affiliations:** 1 Department of Pharmacology, Monash University, Clayton, Victoria, Australia; 2 Anatomy and Developmental Biology, Biomedicine Discovery Institute, Monash University, Clayton, Victoria, Australia; 3 Australian Regenerative Medicine Institute, Monash University, Clayton, Victoria, Australia; 4 Department of Biochemistry and Molecular Biology, Monash Proteomics and Metabolomics Platform, Monash Biomedicine Discovery Institute, Monash University, Clayton, Victoria, Australia; 5 Pathology Queensland at Royal Brisbane and Women’s Hospital, Queensland Health, Queensland, Australia; 6 Novo Nordisk Foundation Center for Stem Cell Medicine, Murdoch Children’s Research Institute, Royal Children’s Hospital, Parkville, Victoria, Australia; 7 Department of Pediatrics, The Royal Children’s Hospital, The University of Melbourne, Parkville, Victoria, Australia; 8 Genetics and Molecular Pathology, South Australia Pathology at Women's and Children's Hospital and Adelaide Medical School and School of Biological Sciences, University of Adelaide, South Australia, Australia; 9 Department of Nephrology, The Royal Melbourne Hospital and Department of Medicine, University of Melbourne, Parkville, Australia

**Keywords:** ALOX15, Fabry nephropathy, Gb3 accumulation, *GLA* variants, induced pluripotent stem cells, α-Gal A deficiency

## Abstract

**Introduction:**

Fabry disease (FD) results from pathogenic *GLA* variants, leading to a deficiency in lysosomal α-galactosidase A (α-Gal A) and accumulation of the sphingolipid globotriaosylceramide (Gb3). This leads to severe renal and cardiovascular complications, primarily affecting kidney podocytes. As a multisystemic disorder, FD initiates at the cellular level; however, the mechanism(s) underlying Gb3-induced cell dysfunction remain largely unknown. This study aimed to identify potential drivers of FD and explore the underlying cell pathology in induced pluripotent stem cell (iPSC)–derived podocytes from patients with FD.

**Methods:**

iPSCs were derived from patients with FD with *GLA*^c.851T>C^ or *GLA*^c.1193_1196del^ variants and compared with controls or CRISPR-Cas9–corrected cell lines. iPSCs were differentiated into podocytes; and α-Gal A activity, Gb3 accumulation, and cell morphology were assessed. Label-free mass spectrometry identified the top, differentially expressed proteins which were validated by using western blot.

**Results:**

Podocytes derived from patients with FD exhibited expression of podocyte-specific markers and morphological features of FD. Reduced α-Gal A activity was observed in FD iPSC-derived podocytes along with the accumulation of Gb3. Proteomic profiling revealed distinct proteomic signatures between control and iPSC-derived podocytes from a patient with FD, with apparent variations among FD lines, highlighting *GLA* variant–specific proteomic alterations. Notably, the ferroptosis-associated protein, arachidonate 15-lipoxygenase (ALOX15), was the most upregulated protein in FD podocytes and ferroptosis was the most enriched pathway. Western blot analysis confirmed the upregulation of ALOX15 in FD podocytes, with validation of other markers implicating ferroptosis in FD pathology.

**Conclusion:**

These findings underscore the heterogeneity of FD and, for the first time, implicate ferroptosis as a potential common pathway driving its pathology.

## Graphical abstract

**Figure:**
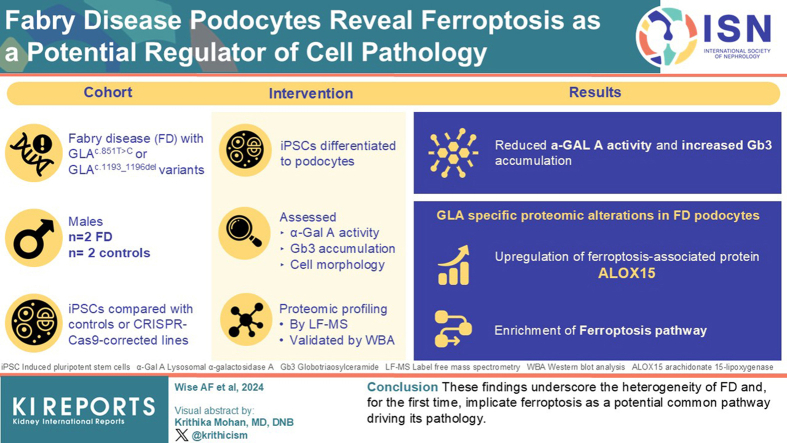

FD is the most common X-linked lysosomal storage disorder worldwide, characterized by pathogenic variants in the *GLA* gene (MIM: 300644) leading to a deficiency in lysosomal α-Gal A. Despite being frequently underdiagnosed, FD manifests across all racial and ethnic backgrounds. In the United States, FD's prevalence is estimated to vary widely, from 1 in 17,000 to 1 in 117,000 males.^1^ In Australia, the current prevalence is approximately 1 in 14,000 live births,^2^ a rate 1.6 times higher than the previously documented rate in 1996,^3^ primarily because of a nearly 10-fold increase in diagnoses.^2^ Patients with FD who have low or absent enzyme activity develop a progressive buildup of the enzyme’s sphingolipid substrate, Gb3, in various affected cells. Notably, Gb3 accumulates in the lysosomes of kidney podocytes, resulting in proteinuria and chronic kidney disease (CKD; Fabry nephropathy), ultimately progressing to end-stage renal disease. Other major clinical manifestations include pain (acroparesthesia), cardiovascular and cerebrovascular disease, gastrointestinal dysfunction, and angiokeratoma, collectively contributing to diminished quality of life and heightened mortality risk.

Podocyte injury is an early hallmark of Fabry nephropathy, where globotriaosylsphingosine (lysoGb3) accumulation contributes to increased extracellular matrix synthesis and fibrosis. Despite our lack of understanding of how Gb3 accumulates within lysosomes, emerging evidence implicates several pathways in podocyte pathophysiology in Fabry nephropathy. Using cultured immortalized podocytes, lysoGb3 was found to activate Notch1 signaling, promoting inflammatory and fibrogenic responses,^4^ while inducing oxidative stress-induced cell death through a RIPK3-dependent pathway.^5^ Notably, Sanchez-Nino *et al.*^4^ demonstrated that inhibiting Notch1 via siRNA or a γ-secretase inhibitor prevented these effects. Similar findings from Fabry kidney biopsies and mouse models underscored the role of Notch1 signaling in the progression of Fabry nephropathy.^4^

Current therapies for FD, including enzyme replacement therapy (ERT) and chaperone-mediated stabilization, aim to reduce intracellular accumulation of Gb3 and mitigate lysosomal dysfunction. However, their ability to reverse kidney injury and CKD remains limited, because replacing functional α-Gal A deficiency does not fully normalize the underlying pathology.^6^ In addition, approximately 40% of treated patients develop antibodies against recombinant α-Gal A, which can neutralize its therapeutic effects, leading to infusion-related responses or anaphylactoid reactions.^7^

Podocyte clearance of Gb3 in FD after ERT is slower and less complete compared with other renal cell types, such as endothelial and mesangial cells.^8^ Despite some reduction in Gb3 accumulation in podocytes after 11 months of ERT, the clearance remains limited, highlighting the challenge of achieving full reversal of glycolipid buildup in these cells.^8^ A recent study using ultrastructural analysis of serial human kidney biopsies showed, that although long-term ERT reduced Gb3 levels in podocytes, it failed to reverse podocyte injury.^9^ In addition, a CRISPR-Cas9–mediated α-Gal A knockout podocyte cell line confirmed the ERT-mediated reduction in Gb3, but without resolving lysosomal dysfunction. Notably, this study identified α-synuclein accumulation as a key driver of podocyte injury, which was resistant to ERT. Genetic and pharmacological inhibition of α-synuclein improved lysosomal function, suggesting that targeting α-synuclein may offer therapeutic benefits beyond Gb3 reduction for patients with Fabry nephropathy.^9^

There remains an urgent unmet need for human experimental models of FD to develop disease modifying therapeutics. This challenge is exacerbated by the absence of accurate disease models that appropriately replicate FD, thereby impeding the development of targeted therapies for Fabry nephropathy and associated complications. Furthermore, it is difficult to maintain primary human podocytes in culture because of their limited and unstable cellular function and viability over extended periods. To address these issues, the derivation of podocytes from iPSCs generated from patients with FD can recapitulate patient-specific genetic variations and phenotypic expressions in an *in vitro* setting. We have previously demonstrated the successful recapitulation of the FD phenotype in cardiomyocytes derived from patient iPSCs and reported a reduction in Gb3 accumulation through CRISPR-Cas9–mediated homology-directed repair.^10^ However, the molecular and cellular phenotype of FD iPSC-derived podocytes has not been previously reported.

In this study, iPSCs were derived from 2 patients with FD, with *GLA*^c.851T>C^ or *GLA*^c.1193_1196del^ variants, both presenting with renal and cardiovascular manifestations, and were compared with controls and isogenic corrected lines^10^ (*GLA*^corr^). iPSCs were differentiated into kidney podocytes^11^, ^12^, ^13^ and α-Gal A activity, Gb3 accumulation, and cell morphology were assessed to confirm the phenotypic features of FD. Label-free mass spectrometry was used to characterize the proteomic changes in iPSC-derived podocytes from patients with FD carrying *GLA*^c.851T>C^ or *GLA*^c.1193_1196del^ variants. This comprehensive analysis revealed distinct proteomic signatures specific to the *GLA* variants, highlighting variant-specific alterations in protein abundance. ALOX15, a key enzyme involved in ferroptotic-cell death emerged as the most significantly increased protein in FD podocytes compared with controls. Moreover, ferroptosis was identified as a common pathway underlying the pathology of FD relative to changes in lysosomal podocyte proteins, with changes in protein markers validated by using western blot analysis. These findings underscore the heterogeneity of FD and, for the first time, implicate ferroptosis as a potential common pathway driving its pathology in the context of lysosomal dysfunction.

## Methods

### Study Participants

The research was carried out in accordance with the Declaration of Helsinki (2008) of the World Medical Association. Ethical approval was obtained from the Royal Melbourne Hospital Human Research Ethics Committee approval 66294/MH-2020 and Monash University Human Research Ethics Committee (Project number CF16/1247 – 2016000663). Informed consent was obtained from all participants.

### Patient Information

In Table 1, the patient information is summarized. Only males were used in the study because of the X-linked inheritance pattern of FD. To ensure consistency and uniformity in the study population and to focus on the classical presentation of FD, which predominantly affects males, 2 male patients presenting with classic FD were selected for inclusion in the study and therefore, 2 male, age-matched control individuals were used for comparison.

**Table 1:** Clinical characteristics of FD patients

Characteristic (normal values)	Fabry *GLA*^c.851T>C^	Fabry *GLA*^c.1193_1196del^
Genders	Male	Male
Age at diagnosis (years)	4	49
*GLA* mutation c	c.851T>C	c.1193_1196del
*GLA* mutation p	p.M284T	p.Gln398fs
Leukocyte a-galactosidase (reference range ≥1.2 nmol/mL/hour)	<0.1	0.2 (truncated protein)
Plasma lysoGb3 (reference range 0.24–0.81 ng/mL)	27 ng/mL	24 ng/mL
Proteinuria (reference range <0.15g/24 h)	2.3g/24h	2.0g/24h
LVH on ECG	Severe	Severe
Angiokeratoma	Yes	Yes
Anhidrosis	Yes	Yes
Chronic neuropathic pain	Yes	No
Brain involvement	Multiple strokes	Stroke and ischemia^a^

ECG, electrocardiogram; FD, Fabry disease; LVH, left ventricular hypertrophy; LysoGb3, globotriaosylsphingosine.

The table shows the key clinical features of patients with Fabry disease having *GLA*^c.851T>C^ or *GLA*^c.1193_1196del^ variants, along with normal values for reference.

a Small white matter lesions and larger ischemic lesions that correlate with their clinical strokes.

### Experimental Design

In this study, iPSCs were derived from patients with FD and healthy controls to model disease-specific cellular behavior. The derivation and culture of these FD iPSCs,^10^ as well as subsequent podocyte differentiation have been published previously,^12^^,^^13^ and are outlined in detail in the Supplementary Methods. In addition, transmission electron microscopy and immunofluorescence microscopy were used to assess cellular ultrastructure and protein expression in these iPSC-derived podocytes, with a comprehensive description of these techniques provided in the Supplementary File. Proteomic and biochemical analyses, including western blotting and measurements of Gb3 and lysoGb3 accumulation, alongside α-Gal A activity assays, were conducted to quantify and compare molecular changes in the cell models, with further methodological details available in the Supplementary Methods. For the measurement of Gb3 and lysoGb3 accumulation, total protein was estimated by the method of Lowry *et al.*^14^ Following proteomics, the raw data files were analyzed with the Fragpipe software suite 17.1^15^^,^^16^ and proteomic data analysis performed using the LFQ-Analyst platform.^17^^,^^18^

### Statistical Analysis

Statistical analyses were performed using Prism v10.2 (GraphPad Software, LLC). Data were presented as the mean ± SEM. Data were identified as outliers if the z-score was more than 3 or less than -3 and subsequently excluded. Experiments were performed a minimum of 3 times in triplicate and significance was calculated with Prism v10.2 (GraphPad Software, LLC) using 1-way analysis of variance and Tukey’s multiple comparison test. A *P* value ≤ 0.05 was considered to indicate statistical significance.

## Results

### Characterization and Differentiation of Podocytes from iPSCs derived from Patients With FD and Controls

iPSCs were derived from dermal skin fibroblasts collected via punch biopsies from male patients with FD, harboring either pathogenic *GLA*^c.851T>C^ or *GLA*^c.1193_1196del^ variants, and from 2 age-matched individuals, who provided informed consent. Both patients with FD presented with renal and cardiovascular disease as indicated by proteinuria and left ventricular hypertrophy, along with a history of stroke (Table 1). Consistent with the diagnosis of FD, both patients exhibited reduced plasma α-Gal A enzyme activity, reflecting the deficiency in α-Gal A enzyme characteristic of the disease. In addition, elevated concentrations of plasma lysoGb3, were observed in both patients (Table 1).

**Figure 1:**
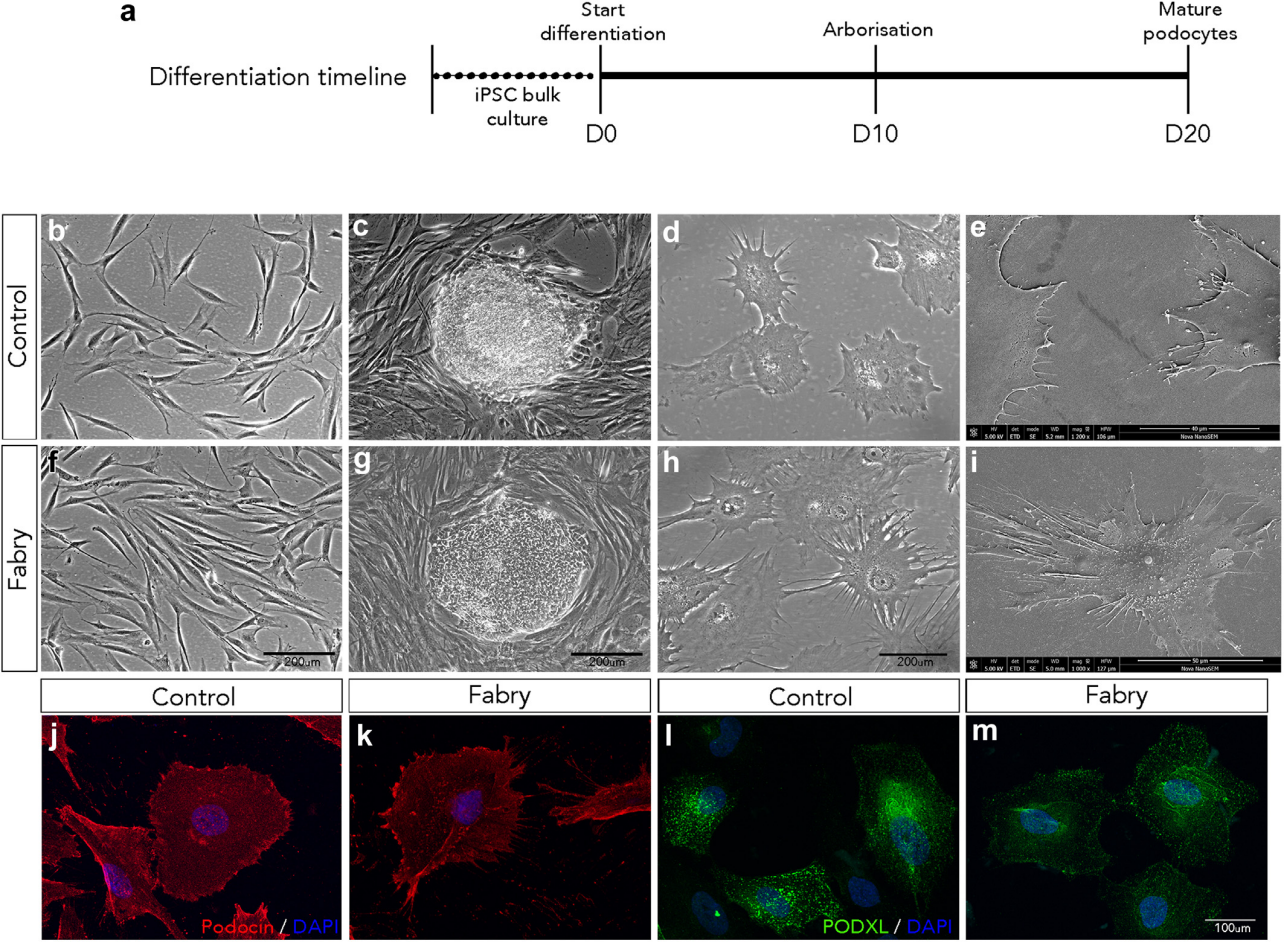
The generation of human iPSC-derived podocytes from patients with FD. (a) A schematic diagram outlining the differentiation timeline of iPSCs into kidney podocytes. Phase contrast microscopy of (b and f) control and patient with FD–derived fibroblasts, (c and g) iPSC colonies, and (d and h) iPSC-derived podocytes following differentiation after 10 days. Scanning electron micrographs of (e) control and (i) FD podocytes displaying a typical podocyte morphology with a cell body evident and protruding primary and secondary cytoplasmic processes after 10 days. Immunofluorescence microscopy showed expression of the podocyte specific markers podocin (red) and podocalyxin (green) in (j and l) control and (k and m) FD podocytes after 10 days. The nuclei were counterstained with DAPI (blue). DAPI, 4’,6-diamidino-2-phenylindole; FD, Fabry disease; iPSCs, induced pluripotent stem cells.

The reprogramming of fibroblasts (Figure 1b and f) to pluripotency (Figure 1c–g) was achieved using mRNA-based overexpression of the Yamanaka factors. Corrected iPSC lines (*GLA*^corr^) were generated through CRISPR-Cas9–mediated homology-directed repair as reported previously.^10^ All cell lines exhibited normal karyotypes and retained expression of pluripotency markers following genetic modification, maintaining stability throughout long-term culture. Podocytes were generated following the directed differentiation up to 20 days (Figure 1a) as previously reported.^11^, ^12^, ^13^ iPSCs from FD and controls differentiated for 10 days, resemble human podocytes that displayed characteristic cytoplasmic processes (Figure 1d and h) and an arborized phenotype evidenced by scanning electron microscopy (Figure 1e and i). Immunofluorescence microscopy confirmed that the differentiated FD and control podocytes after 10 days showed cytoplasmic localization of the podocyte-specific proteins, podocin and podocalyxin (Figure 1j–m).

### Morphological Characteristics of FD in iPSC-Derived Podocytes

**Figure 2:**
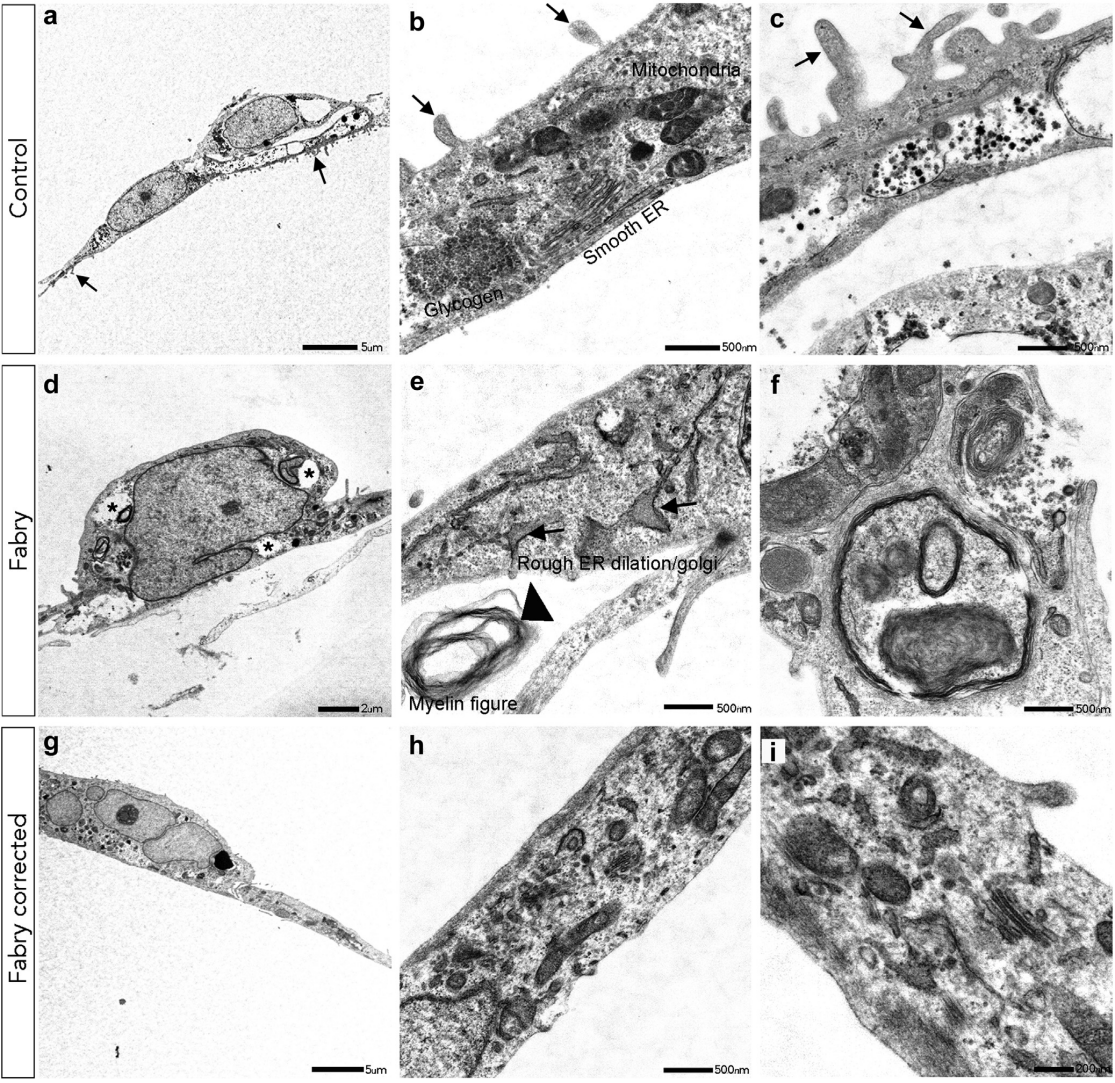
Morphological features of human iPSC-derived podocytes. Representative TEM images of (a–c) control podocytes, (d–f) *GLA*^.c.1193_1196del^ podocytes from patients with FD, and (g–i) *GLA*^corr^ podocytes. In panels a to c, low and high-power images of control iPSC-derived podocytes are presented. (Panel a) At low power, control podocytes exhibit a characteristic cell body and microvilli extensions representing foot processes (arrows). (b and c) High power images show normal cytoplasmic morphology of podocytes including endoplasmic reticulum (ER) and glycogen deposits. (d) In comparison, FD iPSC-derived podocytes showed extensive accumulation of abnormal myelin figures with prominent cytoplasmic vacuoles (asterisks), characteristic of FD. In Panel e, FD podocytes displayed characteristic dilated endoplasmic reticulum (ER; arrows) and myelin abnormalities (large arrowhead). (g–i) Notably, the FD^corr^ podocytes displayed a relatively normal cell morphology with only some lysosomal inclusions evident and no display of abnormal myelin figures. FD, Fabry disease; iPSCs, induced pluripotent stem cells; TEM, transmission electron micrograph.

Transmission electron microscopy was performed to assess the morphological differences in the cell ultrastructure of iPSC-derived podocytes from patients with FD compared with control or *GLA*^corr^ podocytes (Figure 2). Control podocytes exhibited a typical nuclear cell body and cytoplasmic organelles, including glycogen deposits. In addition, podocytes displayed distinct surface microvilli indicative of foot processes (Figure 2a–c). In contrast, FD podocytes displayed notable ultrastructural abnormalities consistent with pathological alterations associated with FD that included the accumulation of abnormal myelin figures and dilated endoplasmic reticulum. Furthermore, ultrastructural abnormalities observed in FD podocytes included vacuoles and lysosomal inclusions (Figure 2d–f). Of significance, *GLA*^corr^ iPSC-derived podocytes exhibited a relatively normal morphology as shown in Figure 2g–i. These cells demonstrated a typical cytoplasmic architecture with no myelin abnormalities identified and only some lysosomal inclusions evident.

### α-Gal A Activity and Gb3 Accumulation in FD Podocytes

**Figure 3:**
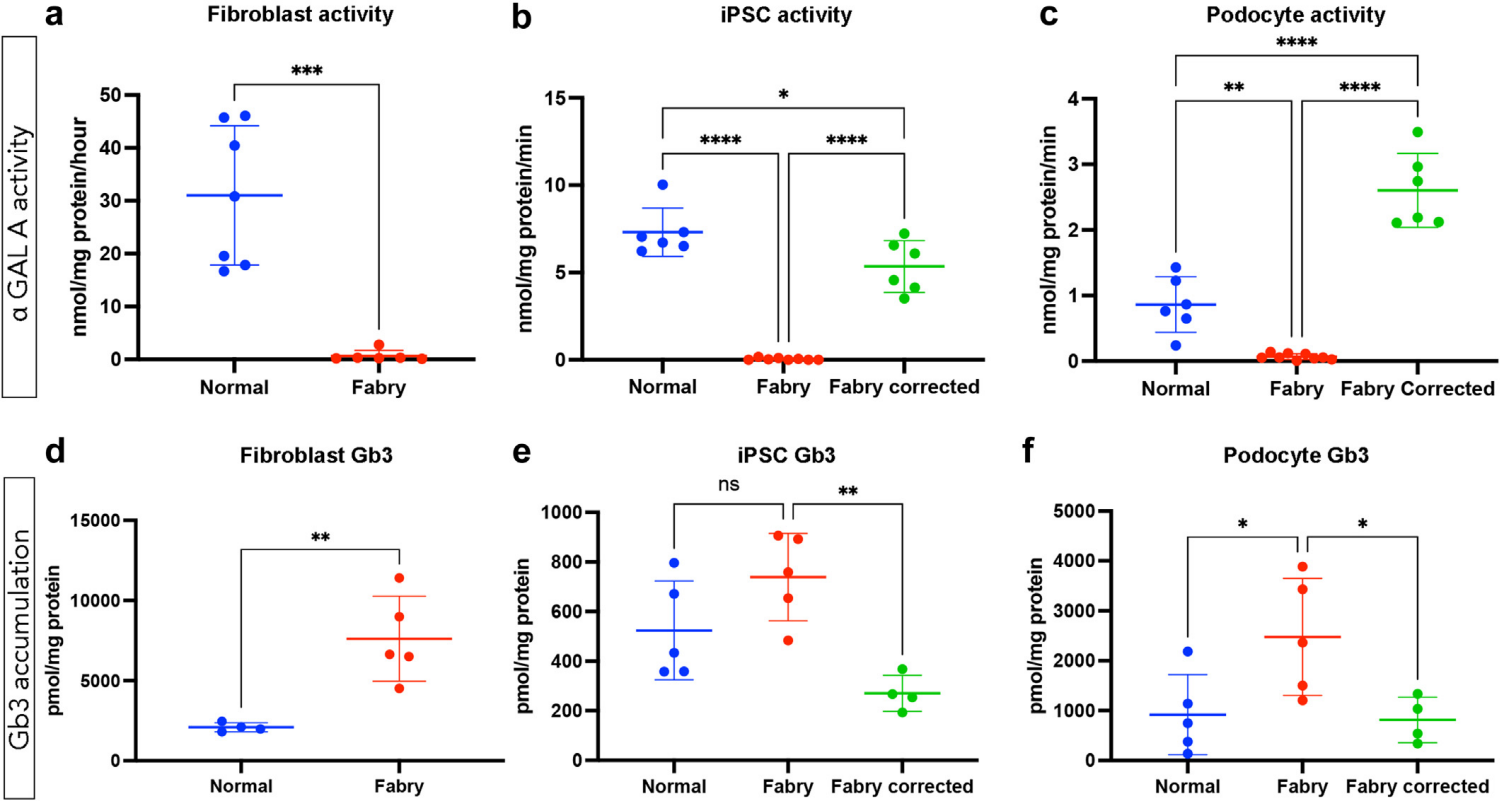
α-galactosidase A activity and Gb3 accumulation in FD podocytes. (a–c) α-galactosidase A activity in fibroblasts, iPSCs, and iPSC-derived podocytes in control, patients with FD with *GLA*^c.851T>C^ or *GLA*^c.1193_1196del^ variants, and FD^corr^ lines. (d–f) Quantification of Gb3 measured by mass spectrometry is also shown. Data are expressed as mean ± SEM. ∗*P* < 0.05. ∗∗*P* < 0.01. ∗∗∗*P* < 0.001. ∗∗∗∗*P* < 0.0001. FD, Fabry disease; Gb3, globotriaosylceramide; iPSCs, induced pluripotent stem cells.

α-Gal A activity was assessed in fibroblasts, iPSCs, iPSC-derived podocytes derived from control individuals, patients with FD carrying *GLA*^c.851T>C^ or *GLA*^c.1193_1196 del^ variants, and *GLA*^corr^ lines. In Figure 3, we demonstrate a significant reduction in α-Gal A activity in FD fibroblasts (*P* < 0.001), FD iPSCs (*P* < 0.0001), and FD podocytes (*P* < 0.01) compared with their respective control counterparts (Figure 3a–c). Notably, a restoration of α-Gal A activity was evident in both FD iPSCs (Figure 3b) and FD podocytes in *GLA*^corr^ lines (*P* < 0.0001; Figure 3c). In addition, the total abundance of Gb3 was quantified using mass spectrometry in fibroblasts, iPSCs and iPSC-derived podocytes derived from control individuals, patients with FD with *GLA*^c.851T>C^ or *GLA*^c.1193_1196del^ variants, and *GLA*^corr^ lines. Consistent with the pathophysiology of FD, elevated Gb3 was observed in both FD fibroblasts (*P* < 0.05) and differentiated podocytes (*P* < 0.05), compared with relevant controls (Figure 3d–f). Following gene correction, there was a significant reduction in Gb3 in *GLA*^corr^ lines, reaching comparable levels in control cells (Figure 3e and f).

### Characterizing Proteomic Variations in FD Podocytes: Insights From Principal Component Analysis and Protein Expression Profiles

**Figure 4:**
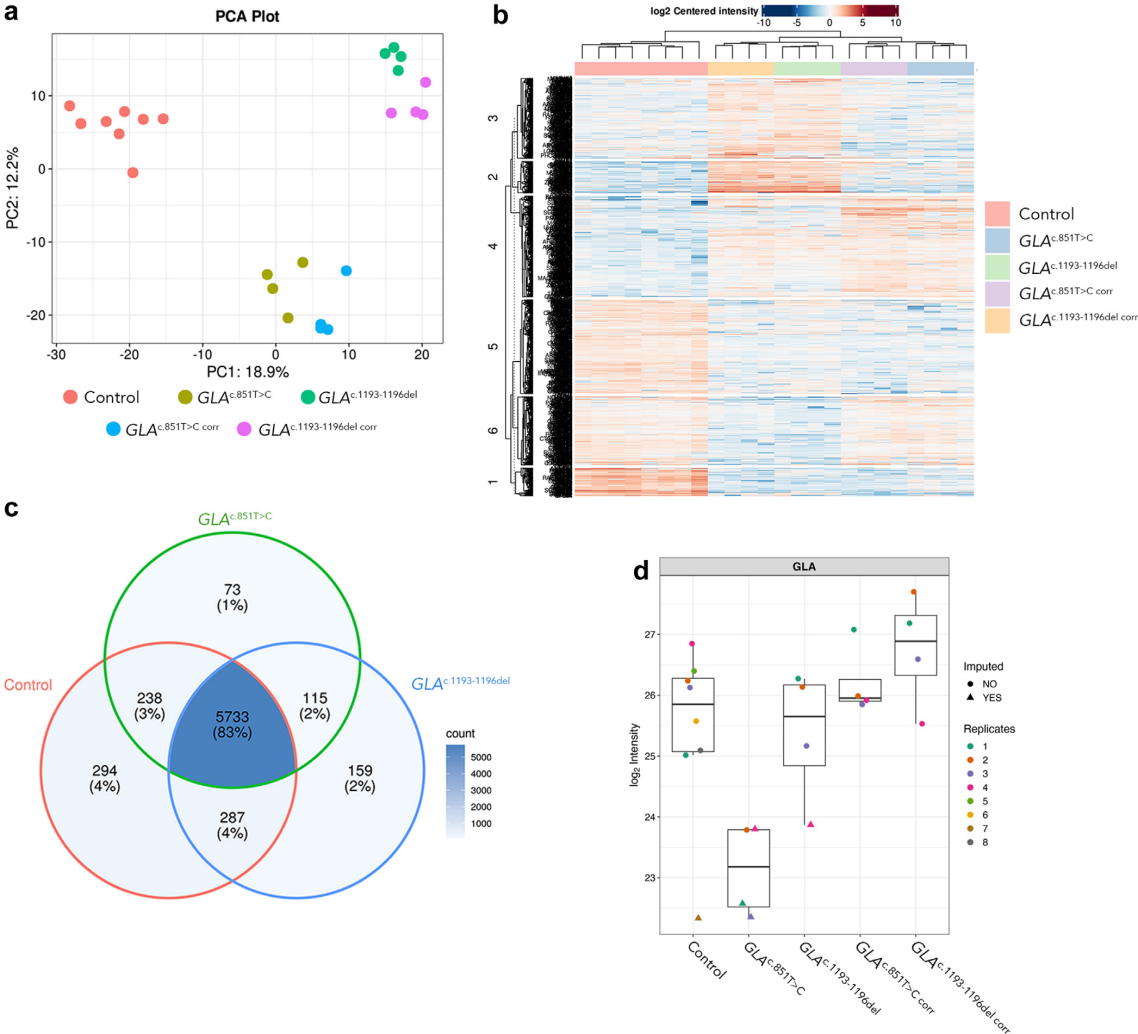
Overview of proteomic analysis of control and FD iPSC-podocytes. (a) Panel a shows principal component analysis (PCA) of control, FD *GLA*^c.851T>C^ or *GLA*^c.1193_1196del^ podocytes and *GLA*^corr^ podocytes. The control groups are represented by red dots which form a tight cluster, indicating homogeneity in proteomic expression. FD podocytes, labeled as *GLA*^c.851T>C^ or *GLA*^1193_1193del^ with their corresponding *GLA*^corr^ podocytes, cluster separately from the control and from each other, highlighting their distinct proteomic profiles. (b) Differential protein heatmap showing a representative overview of significant differentially expressed proteins of FD podocytes compared with controls, as grouped in columns with hierarchical clustering applied. The heatmap indicates the log2-centered intensity levels of proteins, where blue corresponds to lower expression and red to higher expression relative to the mean. (c) The Venn diagram illustrates the overlap of significantly differentially expressed proteins between control, *GLA*^c.851T>C^, and *GLA*^c.1193_1196del^ iPSC-derived podocytes. This visual representation highlights both the shared and unique proteomic changes associated with each Fabry mutation. The overlapping section shows proteins commonly dysregulated in both FD mutations, whereas the nonoverlapping areas represent mutation-specific protein alterations. (d) shows a boxplot of α-Gal A (GLA) abundance for control, *GLA*^c.851T>C^ or *GLA*^c.1193_1196del^ podocytes and their respective *GLA*^corr^ podocytes. The control group shows a narrow range of enzyme levels, with only *GLA*^c.851T>C^ exhibiting significantly lower GLA protein (*P* < 0.05) than the controls, indicating variability because of different mutations and functional protein for each disease line (Table 1 and Supplementary Figure S1). GLA levels for both FD podocytes were comparable with controls following gene correction. Each dot represents an individual measurement of the technical replicates, with the box representing the interquartile range and the whiskers representing the full range of the data. ∗*P*<0.05. FD, Fabry disease; iPSCs, induced pluripotent stem cells.

Principal component analysis was used to simplify the complex proteomic data obtained from the podocytes by condensing it into principal components that capture the most variation (Figure 4a). Principal component 1 represents 18.9% of the variance along the x-axis, whereas principal component 2 represents 12.2% of the variance along the y-axis. The 2 control podocyte lines formed a cohesive cluster, indicating a consistent proteomic profile across biological replicates. Conversely, the *GLA*^c.851T>C^ and *GLA*^c.1193_1196del^ podocytes were distinct from controls but displayed differing proteomic profiles from each other. This suggests that the unique proteomic signatures reflected the distinct mutations present in each cell line.

Proteomics conducted on human podocytes identified 7143 differentially expressed proteins present in the samples, and a protein differential heatmap was generated to visually depict the protein expression profiles (Figure 4b). The heatmap clearly distinguishes the protein abundance patterns of *GLA*^c.851T>C^ and *GLA*^c.1193_1196del^ podocytes compared with control podocytes or *GLA*^corr^ lines and shows significant differences in protein abundance profiles between FD podocytes compared with controls across all clusters. Importantly, within the FD samples, more subtle differences were observed between *GLA*^c.851T>C^ podocytes and *GLA*^c.1193_1196del^ podocytes, suggesting variability in the abundance profiles between the 2 patient-derived lines. These findings align with the observations from the principal component analysis plot.

Given that *GLA*^c.851T>C^ and *GLA*^c.1193_1196del^ podocytes harbored distinct pathogenic variants resulting in the complete absence of α-Gal A in *GLA*^C.851T>C^ and truncated α-Gal A in *GLA*^c.1193_1196del^ (Table 1), proteomics analysis was employed to quantify and compare α-Gal A between these podocytes, and in contrast to *GLA*^corr^ lines. In Figure 4c, we show disparate levels of α-Gal A between *GLA*^c.851T>C^ and *GLA*^c.1193_1196del^ podocytes, compared with control or *GLA*^corr^ lines. *GLA*^c.851T>C^ exhibited significantly lower levels (*P* < 0.05) of α-Gal A compared with the controls, whereas *GLA*^c.1193_1196del^ showed no significant difference. In contrast, both *GLA*^corr^ podocytes showed α-Gal A comparable with controls. Detailed examination of α-Gal A peptide intensity for each patient with FD (Supplementary Figure S1) confirmed the detection of only 1 peptide in *GLA*^c.851T>C^ podocytes (peptide 7: 2.05 × 10^6^), whereas 2 peptides (peptide 6: 3.29 × 10^7^ and peptide 7: 5.71 × 10^7^) were detected in *GLA*^c.1193_1196del^ podocytes. In contrast, control cell lines exhibited 3 to 4 peptides (Supplementary Figure S1). This disparity observed between the FD podocyte lines underscores the substantial variation in α-Gal A even within the same disease classification.

### ALOX15: A Key Protein in the Ferroptosis Pathway for FD Podocytes

**Figure 5:**
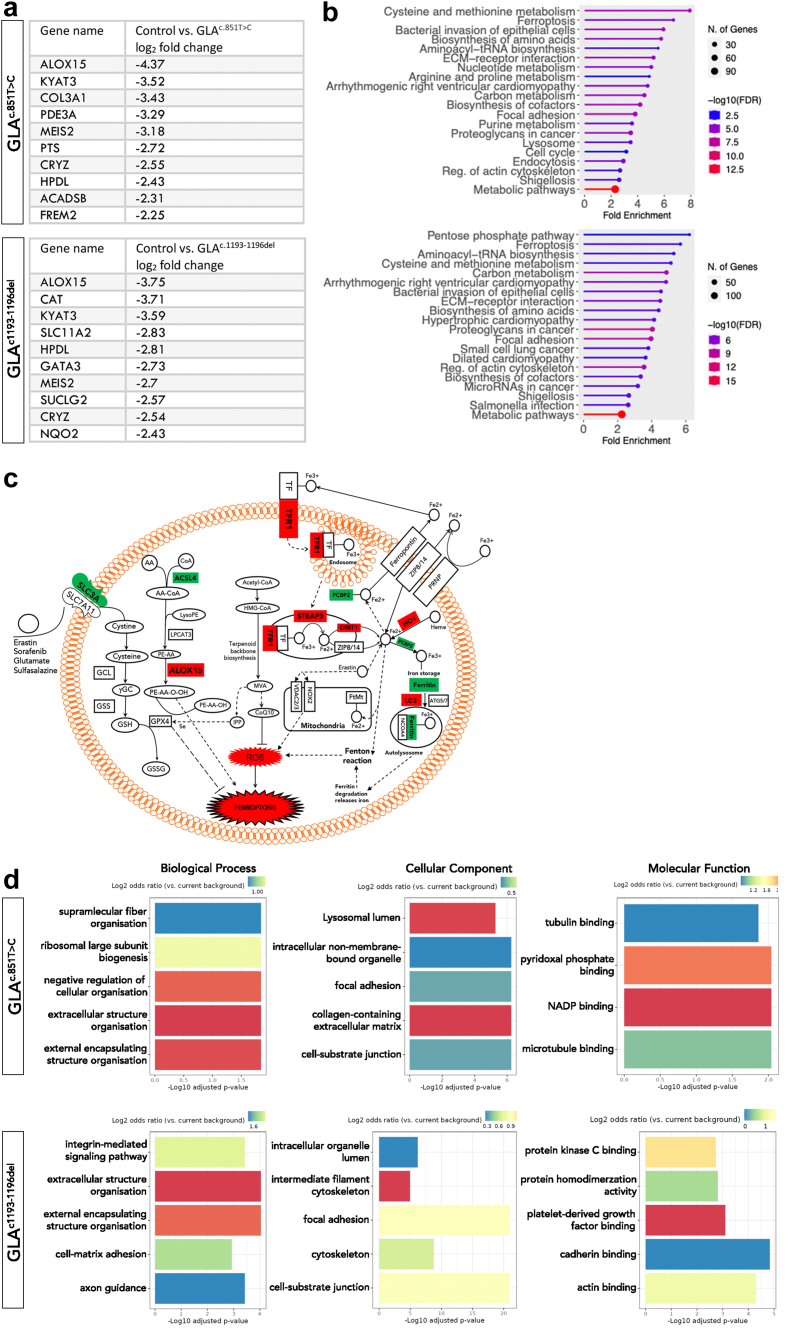
Comparative proteomic analysis and ferroptosis enrichment in FD podocytes. Proteomic enrichment analysis utilizing LFQ-Analyst and ShinyGO revealed significant insights into the protein abundance profiles of *GLA*^c.851T>C^ or *GLA*^c.1193_1196del^ podocytes. (a) The top 10 proteins of increased abundance, with ALOX15 emerging as a common top upregulated protein in *GLA*^c.851T>C^ (top panel) and *GLA*^c.1193_1196del^ (lower panel) podocytes. (b) Pathway enrichment analysis based on KEGG highlighted ferroptosis as a prominent pathway enriched in FD podocytes compared with controls. (c) A schematic diagram of ferroptosis component quantification for FD podocytes compared with controls, including ACSL4, Ferritin, SLC3A, TRF1, STEAP3, DMT1, HO1, PCBP2, and LC3. Differential protein abundance is denoted for red indicating an increase and green a decrease, relative to controls. (d) GO enrichment plots depict the top 5 enriched terms in *GLA*^c.851T>C^ or *GLA*^c.1193_1196del^ podocytes compared with controls, categorized by biological processes, cellular components, and molecular functions. The log2 odds ratio scale shown at the top of the image represents the relative enrichment of each GO term (biological process, cellular component, or molecular function) in the data set being analyzed compared with the background. The scale range indicates different levels of enrichment. ACSL4, acyl-CoA synthetase long-chain family member 4; ALOX15, arachidonate 15-lipoxygenase; DMT1 divalent metal transporter 1; FD, Fabry disease; GO, gene ontology; HO1, heme oxygenase 1; KEGG, Kyoto Encyclopedia of Genes and Genomes; LC3, microtubule-associated proteins 1A/1B light chain 3B; PCBP2, poly(C)-binding protein 2; SLC3A, solute carrier family 3 member 2; STEAP3, 6-transmembrane epithelial antigen of the prostate 3; TRF1, telomeric repeat binding factor 1.

Comparative proteomics and enrichment analysis were conducted utilizing the bioinformatics platforms LFQ-Analyst and ShinyGO to compare each protein identified. The comparative analysis revealed significant differences in 1173 of the 7143 proteins detected between the control and *GLA*^c.851T>C^ and *GLA*^c.1193_1196del^ podocytes. This extensive variation in protein expression suggests a significant impact of FD on cellular protein expression in podocytes. Among these differentially abundant proteins, ALOX15, a key regulator of ferroptosis, emerged as the common increased abundant protein in both *GLA*^c.851T>C^ and *GLA*^c.1193_1196del^ podocytes (Figure 5a). Furthermore, pathway enrichment analysis identified ferroptosis as the common top dysregulated pathway in both *GLA*^c.851T>C^ and *GLA*^c.1193_1196del^ podocytes, following cysteine-methionine metabolism in *GLA*^c.851T>C^ podocytes and the pentose phosphate pathway in *GLA*^c.1193_1196del^ podocytes (Figure 5b). The increased significance of ferroptosis and ALOX15 in *GLA*^c.851T>C^ and *GLA*^c.1193_1196del^ podocytes implies potential shared mechanism(s) in the pathophysiology of FD. As depicted in the summary schematic (Figure 5c), 9 additional proteins associated with the ferroptosis pathway were differentially abundant in both *GLA*^c.851T>C^ and *GLA*^c.1193_1196del^ podocytes. These proteins include acyl-CoA synthetase long-chain family member 4 (ACSL4), ferritin, solute carrier family 3 member 2, telomeric repeat binding factor 1, 6-transmembrane epithelial antigen of the prostate 3, divalent metal transporter 1, heme oxygenase 1, poly(rC)-binding protein 2, and microtubule-associated proteins 1A/1B light chain 3B. Among these proteins, 4 (ACSL4, ferritin, solute carrier family 3 member 2, and poly(rC)-binding protein 2) showed decreased abundance, whereas the remaining proteins showed an increase. In addition, Gene Ontology enrichment analysis revealed the involvement of lysosomes in both *GLA*^c.851T>C^ and *GLA*^c.1193_1196del^ podocytes (Figure 5d). The differentially abundant proteins were mainly involved in the regulation of organelles and their lumen, cell shape, cell communication, extracellular organization, and various signaling activities, reflecting the impact of lysosomal dysfunctions inherent in FD. A network map of the enriched biological pathways is presented in Supplementary Figure S2.

### Validation of Ferroptosis Protein Markers in Podocytes: Western Blot Analysis of ALOX15, Ferritin, and ACSL4 Expression

**Figure 6:**
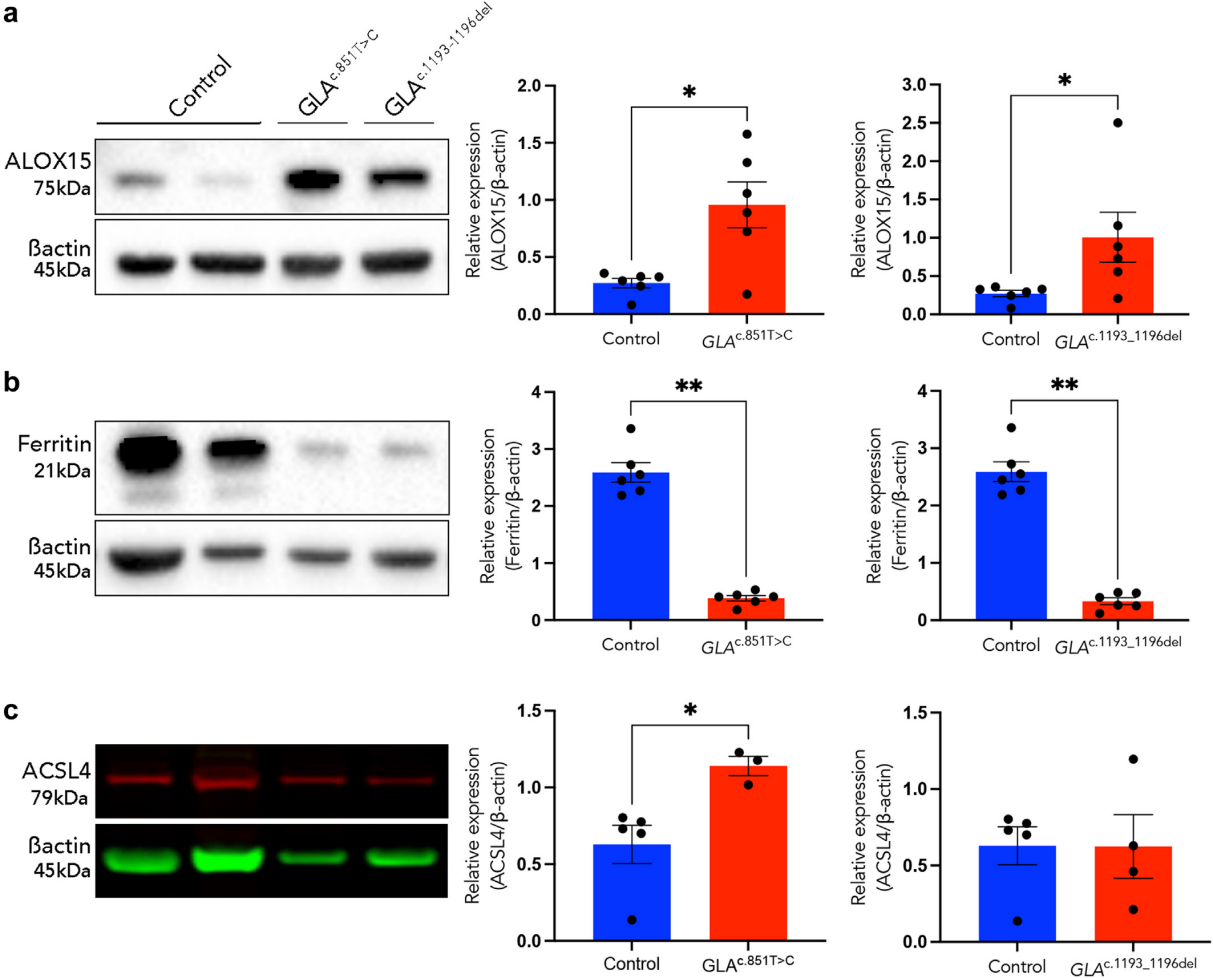
Differential expression of ferroptosis-related proteins in control and FD podocytes. Western blot analysis was performed to quantify relative protein expression normalized to β-actin, with (a–c) western blot images illustrating the expression levels of ALOX15, ferritin, and ACSL4 alongside β-actin protein levels in both control podocytes and *GLA*^c.851T>C^ or *GLA*^c.1193_1196del^ podocytes. (a) ALOX15 expression was significantly higher in both *GLA*^c.851T>C^ or *GLA*^c.1193_1196del^ podocytes (*P* < 0.05). (b) Ferritin expression was significantly lower in both *GLA*^c.851T>C^ and *GLA*^c.1193_1196del^ podocytes (*P*<0.05). (c) ACSL4 expression was significantly higher in *GLA*^c.851T>C^ podocytes (*P* < 0.05), whereas no difference was observed in *GLA*^c.1193_1196del^ podocytes. Mean ± SEM and each point corresponds to a technical replicate. ∗*P* < 0.05. ACSL4, acyl-CoA synthetase long-chain family member 4; ALOX15, arachidonate 15-lipoxygenase; FD, Fabry disease.

Validation of ferroptosis-associated protein markers ALOX15, ferritin, and ACSL4 was conducted by using western blot analysis in *GLA*^c.851T>C^ and *GLA*^c.1193_1196del^ podocytes and compared with controls (Figure 6). A statistically significant increase in ALOX15 expression was observed in podocytes compared with controls (Figure 6a; *P* < 0.05). Likewise, ferritin expression was significantly lower (P < 0.05) in both *GLA*^c.851T>C^ and *GLA*^c.1193_1196del^ podocytes compared with controls (Figure 6b). Moreover, ACSL4 expression showed a significant increase in *GLA*^c.851T>C^ podocytes compared with controls (*P* < 0.05), whereas no significant difference was observed in *GLA*^c.1193_1196del^ podocytes (Figure 6c). Notably, GPX4 expression remained unchanged between control and FD podocytes (data not shown). The significantly altered expression of ALOX15 and ferritin in FD podocytes irrespective of pathogenic variant aligns with the profile of ferroptosis, characterized by reduced iron storage.

## Discussion

This study used proteomics to identify potential drivers of FD and explore the underlying cellular pathology in iPSC-derived podocytes from patients with FD with *GLA*^c.851T>C^ or *GLA*^c.1193_1196del^ variants who presented with renal and cardiovascular symptoms. Label-free mass spectrometry identified the top differentially abundant proteins, which were subsequently validated by using western blot analysis. Podocytes derived from patient-derived iPSCs displayed morphological features of FD, alongside reduced α-Gal A activity and Gb3 accumulation. Proteomic profiling revealed distinct protein signatures between control and FD podocytes, highlighting *GLA* variant-specific alterations. Notably, ALOX15, a protein associated with ferroptosis, emerged as the most significantly increased protein, and ferroptosis as a commonly enriched pathway in FD podocytes compared with controls. Interestingly, ferritin levels were reduced in FD podocytes, suggesting a dysregulation of iron metabolism. This study highlights the importance of understanding the impact of *GLA* variants on cellular pathways using iPSC-derived models for advancing therapeutic strategies for FD treatment. Moreover, it underscores the novel exploration of iron-dependent ferroptosis, which may be intricately linked to lysosomal dysfunction in FD, with proteins such as ALOX15 or ACSL4 potentially mediating this process.

In addition to proteomic studies of patient plasma that identified inflammatory and angiogenesis-related proteins,^19^^,^^20^ our analysis of Fabry iPSC-derived podocytes offers novel insights into disease-associated biological processes. This complements previous findings where plasma biomarkers such as lysoGb3 were inconsistently elevated, particularly in milder forms of FD and in some female patients.^20^ Althoughthe accumulation of glycosphingolipids because of α-Gal A deficiency is the primary driver of FD, secondary dysfunctions at the cellular, tissue, and organ levels contribute significantly to disease progression. Tebani *et al.*^21^ conducted a large-scale plasma proteomic profiling of deeply phenotyped patients with FD (*n* = 55) and identified 615 differentially expressed proteins, of which 365 were newly reported, including key players in cytokine-mediated pathways, extracellular matrix remodeling, and lysosomal function. Proteins such as CD200, NOTCH1, and GRN were implicated in proinflammatory responses and extracellular matrix remodeling.

Our study also identified ferroptosis, a form of regulated cell death characterized by iron-dependent lipid peroxidation, as a potential contributor to disease pathology. Initially reported by Dixon *et al.*,^22^ ferroptosis has recently garnered attention in the context of kidney disease and diabetes.^23^, ^24^, ^25^, ^26^, ^27^, ^28^, ^29^ Ischemia-reperfusion injury, a common form of acute kidney injury, has been associated with iron-dependent ferroptotic cell death in renal tubular epithelial cells.^25^, ^26^, ^27^^,^^29^, ^30^, ^31^ Furthermore, there is emerging evidence implicating ferroptosis in CKD disease progression.^32^ Diabetic nephropathy, a common complication of diabetes and leading cause of CKD, has been linked to ferroptosis.^33^^,^^34^ In addition, ferroptosis-related molecules were detected in kidney biopsies of patients with diabetic nephropathy and was associated with tubular cell injury and oxidant stress in diabetic mouse models.^34^

The recent discovery of the role of autophagy in the induction of ferroptosis^35^ may shed new light on lysosomal disorders such as FD and suggests a broader relevance beyond renal tissue. A connection between iron overload, increased lipid peroxidation, and lysosomal involvement has been reported,^36^^,^^37^ suggesting a potential link to ferroptosis. These pathways have prompted a revaluation of disturbances in iron metabolism and lipid peroxidation as factors in the pathogenesis of lysosomal disease, viewed through a new lens of ferroptosis involvement (for review^38^). Building on these earlier findings, it has become evident that disturbances in iron metabolism and lipid peroxidation may contribute to the pathogenesis of lysosomal disease.^25^^,^^37^ The relationship between iron overload, lipid peroxidation, and lysosomal involvement underscores the need for a comprehensive understanding of these processes in the context of disease progression, including FD, which may better inform potential therapeutic targets.

Our study has shown that ferroptosis and lysosomal pathways were notably enriched in *GLA*^c.851T>C^ and *GLA*^c.1193_1196del^ podocytes, compared with both control and corrected lines. Specifically, the expression of ALOX15, a protein associated with ferroptosis, was significantly upregulated in FD podocytes compared with controls as confirmed by western blot analysis. Chien *et al.*^39^ previously identified increased expression of arachidonate 12/15-lipoxygenase in iPSC-derived cardiomyocytes from patients with FD with the IVS4+919 G>A variant, which is linked to a late-onset cardiac phenotype. Moreover, the inhibition of ALOX15 in iPSC-derived cardiomyocytes was found to improve the efficacy of α-Gal A to ameliorate cardiomyocyte hypertrophy when administered early in culture.^39^ Another ferroptosis marker identified in FD podocytes was ACSL4, involved in the biosynthesis of long-chain polyunsaturated fatty acids, particularly arachidonic acid and adrenic acid, which serve as substrates for the generation of lipid peroxides.^28^ Our study showed that ACSL4 was upregulated in FD podocytes from the *GLA*^c.851T>C^ variant, further supporting the involvement of ferroptosis in FD pathology. In support of this data, the inhibition of ACSL4-driven ferroptosis has been shown to rescue from angiotensin II–induced renal injury^40^ and the development of fibrosis because of unilateral ureteral obstruction^41^ or ischemic acute kidney injury^42^ in mice. In addition, microtubule-associated protein 1A/1B-light chain 3, a protein involved in autophagosome-lysosome fusion, was found to have decreased abundance in FD podocytes. In the context of lysosomal storage diseases, the role of microtubule-associated protein 1A/1B-light chain 3 is primarily related to the clearance of accumulated cellular waste and damaged organelles, including dysfunctional lysosomes.^43^

The observed reduction of ferritin levels in *GLA*^c.851T>C^ or *GLA*^c.1193_1196del^ podocytes may be attributed to dysregulated iron metabolism, lysosomal dysfunction, increased oxidative stress, and ferroptosis activation. These factors collectively disrupt iron homeostasis, impairing the cells’ ability to sequester iron effectively. Using a computational transcriptomics approach. Delaleu *et al.*^44^ identified lower transferrin receptor protein synthesis in glomeruli and its ligand, transferrin, which were significantly downregulated in arteries of patients with FD on 10 years of ERT compared with healthy controls. Serum ferritin levels have been recognized as an indicator of inflammatory disease and declining renal function,^45^^,^^46^ likely because of its release from damaged cells.^47^ Dysregulated ferritin levels have also been observed in kidney diseases characterized by podocyte injury (for review^48^). In addition, an accumulation of iron in kidney proximal tubular epithelial cells has been linked to ferroptosis,^30^ and associated with lipid peroxidation in tubular cells during ischemic injury.^31^ Studies in other systems, such as neurological disease^49^ and cancer,^50^ suggest ferritin's protective role against ferroptosis, and its degradation can increase labile iron pool accumulation, further promoting ferroptosis. Dysregulated iron metabolism and oxidative stress are evident in these conditions; however, ferritin's specific involvement in podocyte injury and the progression of disease remains unclear.

One limitation of the present study is the derivation of iPSCs exclusively from male patients with FD and control individuals, that stems from the X-linked nature of the disease.^51^ FD is recognized as a relentlessly progressive disorder with significant heterogeneity in its clinical manifestations.^52^ Contrary to earlier perceptions that heterozygous females were asymptomatic carriers, recent studies show a higher prevalence of affected women than men.^2^ Moreover, it has become increasingly evident that women harboring a single copy of a pathogenic *GLA* variant are at a significant risk of suffering multisystemic disease with reduced quality of life.^25^^,^^53^ Although females are often reported to develop CKD later than males, Schiffmann *et al.*^54^ found a similar median age at dialysis initiation for both genders (42.4 years in women vs. 39.5 years in men). In addition, Thadhani *et al.*^55^ observed that 88% of patients with Fabry starting dialysis were male, with only 12% female, suggesting underrepresentation of females in advanced CKD despite the expected 1:2 male-to-female ratio for this X-linked disease.

Understanding how *GLA* variants disrupt crucial pathways is imperative for developing therapeutic interventions and holds promise for enhancing diagnostic and therapeutic strategies tailored to the diverse clinical needs of affected individuals. By employing human iPSC disease modeling, this study elucidates the complex molecular mechanisms implicated in FD pathophysiology, providing fresh insights into potential therapeutic targets. The consistent findings observed using 2 pathogenic FD variants, *GLA*^c.851T>C^ and *GLA*^1193_1196del^, with their differing proteomic signatures and mutations, emphasize the significance of considering variant-specific alterations in FD research. The study underscores ferroptosis as a potential therapeutic target for future interventions aimed at managing or preventing complications associated with FD.

## Disclosure

KMN received research grant support and speaker/advisory board honoraria from Shire/Takeda, Sanofi/Genzyme. All the other authors declared no competing interests.

## Supplementary Material

Supplementary File (PDF) **Supplementary Methods. Figure S1.** Variation in α-Gal A peptide intensity highlights heterogeneity within FD podocytes. **Figure S2.** Network Map of Enriched Biological Pathways in Proteomics Analysis.

## Footnotes

Supplementary File (PDF)

**Supplementary Methods.**
**Figure S1.** Variation in α-Gal A peptide intensity highlights heterogeneity within FD podocytes. **Figure S2.** Network Map of Enriched Biological Pathways in Proteomics Analysis.
