# Supplementary material for: Fabry Disease Podocytes Reveal Ferroptosis as a Potential Regulator of Cell Pathology
Source: Kidney Int Rep. 2024 Nov 23;10(2):535–48. doi: 10.1016/j.ekir.2024.11.024 (PMC11843119; doi:10.1016/j.ekir.2024.11.024)
Supplement: Supplementary File (PDF) — Supplementary Methods. Figure S1. Variation in α-Gal A peptide intensity highlights heterogeneity within FD podocytes. Figure S2. Network Map of Enriched Biological Pathways in Proteomics Analysis. [file mmc1.pdf]

A Alpha-galactosidase A (429 residues)

```
1-50    MQLRNPELHL GCALALRFLA LVSWDIPGAR ALDNGLARTP TMGWLHWERF
51-100  MCNLDCQEEP DSCISEKLFM EMAELMVSEG WKDAGYEYLC IDDCWMAPQR
101-150 DSEGRLQADP QRFPHGIRQL ANYVHSKGLK LGIYADVGNK TCAGFPGSFG
151-200 YYDIDAQTFA DWGVDLLKFD GCYCDSLENL ADGYKHMSLA LNRTGRSIVY
201-250 SCEWPLYMWP FQKPNYTEIR QYCNHWRNFA DIDDSWKSIK SILDWTSFNQ
251-300 ERIVDVAGPG GWNDPDMLVI GNFGLSWNQQ VTQMALWAIM AAPLFMSNDL
301-350 RHISPQAKAL LQDKDVIAIN QDPLGKQGYQ LRQGDNFEVW ERPLSGLAWA
351-400 VAMINRQEIG GPRSYTIAVA SLGKGVACNP ACFITQLLPV KRKLGFYEWT
401-429 SRLRSHINPT GTVLLQLENT MQMSLKDLL
```

Peptide sequence
- Peptide 7 TPTMGWLHWER
- Peptide 4 QLANYVHSK
- Peptide 5 QYCNHWR
- Peptide 3 NFADIDDSWK
- Peptide 6 SILDWTSFNQER
- Peptide 1 KLGFYEWTSR
- Peptide 2 LGFYEWTSR

B

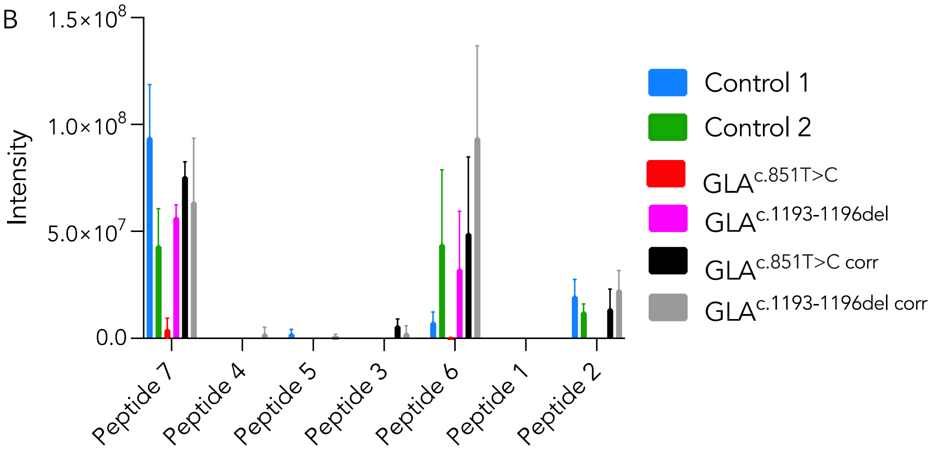

Supplementary Figure 1: Variation in α-Gal A peptide intensity highlights heterogeneity within FD podocytes. Analysis of α-Gal A peptide intensity for FD patients with *GLA*$^{c.851T>C}$ or *GLA*$^{c.1193\_1196del}$ variants demonstrated the presence of only one peptide in *GLA*$^{c.851T>C}$ podocytes (peptide 7: 2.05 x 10^6), while *GLA*$^{c.1193\_1196del}$ podocytes displayed two peptides (peptide 6: 3.29 x 10^7 and peptide 7: 5.71 x 10^7). Conversely, control cell lines exhibited a higher diversity with three to four peptides detected.

Supp Figure 2

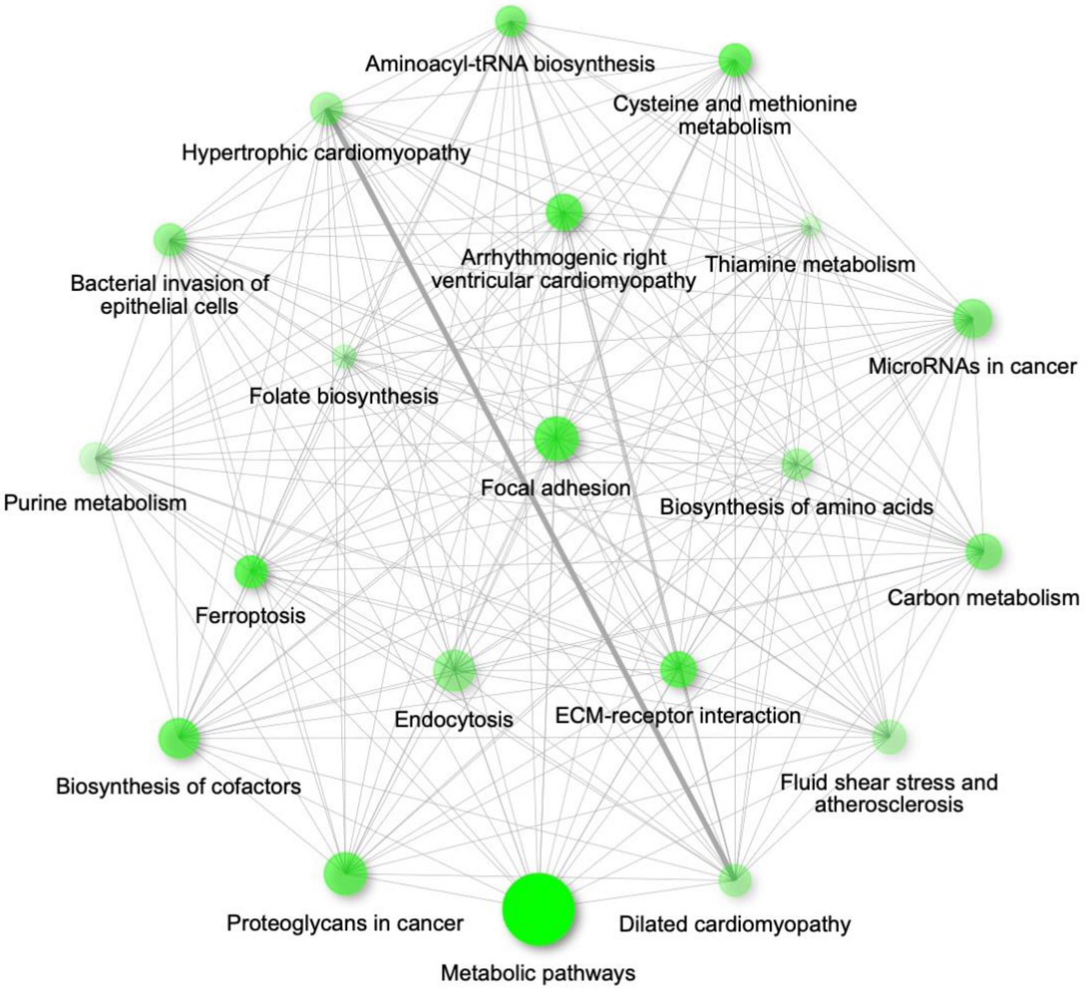

Supplementary Figure 2: Network Map of Enriched Biological Pathways in Proteomics Analysis. This figure depicts a network of enriched biological pathways identified in the proteomics analysis between control and FD $\textit{GLA}^{c.851T>C}$ or $\textit{GLA}^{c.1193\_1196del}$ podocytes. Nodes represent pathways, with larger nodes indicating higher significance. Edges show interactions between pathways, with thicker lines representing stronger associations. Key pathways include "Metabolic pathways," "Focal adhesion," and "Dilated cardiomyopathy." This map highlights the complex interplay between various biological processes.

Derivation and culture of iPSCs

Human dermal fibroblasts were isolated from 4-6mm skin punch biopsies from male FD patients with mapped gene mutations, and control individuals who were confirmed to have normal plasma globotriaosylsphingosine (lysoGb3) levels and α-Gal A activity. Fibroblasts were cultured in DMEM medium supplemented with 10% fetal bovine serum (FBS), 1% L-glutamine (200mM), 1% penicillin-streptomycin (10,000 U/mL) and 0.1% fungizone. iPSCs were generated from the human fibroblasts using the stemRNA-NM reprogramming kit (Reprocell, USA) according to the manufacture's guidelines. On day 10-14 following transfection, the newly-formed iPSC colonies were mechanically dissected and replated on either mouse embryonic fibroblasts with ES medium (DMEMF/12 medium supplemented with 20% knockout serum replacement, non-essential amino acid (NEAA; 10mM) and 10ng/µl basic fibroblast growth factor or Geltrex matrix with Essential 8 (E8) medium and incubated at 37°C in 5% CO_2. Once established, all lines were subsequently cultured on Geltrex matrix in E8 medium, with medium changed every 1-2 days. All lines were pluripotent and exhibited a normal karyotype.

Podocyte differentiation

To induce podocyte differentiation, when iPSCs had reached 50% confluency, the E8 medium was removed, and the flask rinsed with phosphate buffered saline (PBS). To prepare podocytes for western blot quantification, podocyte differentiation was started at 70-80% confluency in order to obtain a higher cell count for the assay. Podocyte differentiation medium, consisting of DMEM/F12 supplemented with 2.5% FBS, 1% 10mM NEAA, 1% penicillin-streptomycin (10,000 U/mL), 10ng/mL activin A, 15ng/mL bone morphogenic protein 7 (BMP7) and 0.1M retinoic acid, was added. Medium was changed every alternate day for 10 days. Additionally, 10 mM Y-27632 (rho-associated protein kinase inhibitor or ROCK inhibitor) was added when plating the podocytes after splitting to promote enhanced attachment. For long term culture of iPSC-derived podocytes, cells were maintained in podocyte medium without the addition of activin A, BMP7 and retinoic acid.

Transmission electron microscopy (TEM)

Differentiated podocytes were isolated and fixed in Karnovsky's fixative and washed in 1 mL 0.1M cacodylate buffer before post-fixation in 1% osmium tetroxide for one hour at room temperature. Serial dehydration was performed before embedding in resin. Pellets were thin sectioned with an ultra-microtome and placed in copper orthogonal grids before staining with 4% uranyl acetate. All sections were imaged using a Hitachi H7500 TEM (Hitachi) or Tecnai T12 TEM (FEI) with Gatan Microscopy Suite Software (Gatan Incorporated).

Immunofluorescence microscopy

Immunofluorescence staining of iPSC-derived podocytes was performed as previously described [2]. In brief, cells were serum starved overnight, fixed with 4% paraformaldehyde for 10 min, permeabilized with 0.2% Triton X-100 for 10 min and blocked with 5% bovine serum albumin for 1 hour. Cells were stained with primary antibodies anti-podocin (Abcam) or anti-podocalyxin (R&D Systems) in blocking solution overnight at 4°C followed by secondary antibodies (Alexa Fluor 488 and 555; Life Technologies) in PBS for 1 hour. Cells were counterstained with 4',6-diamidino-2-phenylindole (DAPI; Life Technologies) and then mounted with fluorescent mounting medium (DAKO). Staining was imaged with a Stellaris 5 confocal (Leica) microscope.

Western Blot analysis

Cells were lysed with radioimmunoprecipitation assay (RIPA) lysis buffer (Pierce™ Thermo Fisher Scientific for acyl-CoA synthetase long-chain family member 4 (ACSL4; Cell Signaling for ALOX15 and Ferritin) containing a protease inhibitor (Sigma-Aldrich), and protein concentration was determined using the Pierce™ BCA protein assay kit (Thermo Fisher Scientific). Samples for ACSL4 were denatured for 10 minutes at 95°C, while those for ALOX15 and Ferritin were boiled for 5 minutes. Subsequently, all samples underwent sodium dodecyl sulfate-polyacrylamide gel electrophoresis (SDS-PAGE). Gels were hand-cast for ALOX15 and Ferritin using Bio-Rad solutions and run with Tris/Tricine/SDS running buffer (Bio-Rad) at 80V for 30 minutes and followed by 150V for 1 hour. Pre-cast Bolt™ 4-12% Bis-Tris Plus Gels (Thermo Fisher Scientific) were used for ACSL4, run at 200V for 22 minutes. Proteins were transferred to nitrocellulose membranes using different conditions: for ALOX15 and Ferritin, a Bio-Rad Trans-Blot Turbo Transfer System was used. For ACSL4, transfer was achieved at a lower voltage of 10V for 60 minutes. Membranes for ACSL4 were blocked using Intercept® blocking buffer (LI-COR) for 1 hour at room temperature. For ALOX15 and Ferritin, membranes were blocked with 7% non-fat milk in Tris-buffered saline containing 0.5% Tween® 20 detergent overnight at 4°C to reduce non-specific binding. Primary antibodies were applied overnight at 4°C: ACSL4 (1:10,000; Clone EPR8640; Abcam), ALOX15 (1:500; Clone EPR22138; Abcam) and Ferritin (1:500; Clone 8H10D10; Abcam), with β-actin (1:1000; Clone mAbcam 8224; Abcam) as the loading control. Visualization for ACSL4 was conducted using the Odyssey CLX (LI-COR) with IRDye conjugated secondary antibodies. For ALOX15 and Ferritin, a standard chemiluminescence (ECL) kit (Bio-Rad) was used. The intensity of protein bands was quantified relative to β-actin using Image Studio 5.2 software (LI-COR) for ACSL4 and Image Lab 6.1.0 software (Bio-Rad) for ALOX15 and Ferritin. Each sample was run in triplicate.

Measurement of Gb3 and lysoGb3 accumulation and α-Galactosidase A Activity Assay

Confluent fibroblasts, iPSCs and iPSC-derived podocytes were harvested as cell pellets in triplicate and homogenized by sonication (Misonix, Farmingdale, NY) in 0.02 M Tris (containing 0.5 M NaCl and 0.1% v/v Nonidet P-40; pH 7.0). Lipids were extracted from 50 μg of protein in 10 μL of homogenate

using a single-phase lipid extraction by the addition of 0.2 mL chloroform/methanol (2/1) and 5 µL of internal standard (IS) mixture containing 10 pmol THC (d18:1/17:0) [*N*-heptadecanoyl ceramide trihexoside] from Matreya LLC (State College, PA). Samples were mixed for 10 min, sonicated for 30 min, and allowed to stand at RT for 20 min. Samples were then centrifuged (13000 × *g*; 10 min at RT) and the supernatant was removed and dried down under N_2 at 40°C. Lipid extracts were reconstituted in 10 mM ammonium formate in methanol, and partial separation was achieved on an Agilent Zorbax Eclipse C18 column (2.1 × 50 mm, 1.8 µm) maintained at 40 °C with a flow rate of 0.4 mL/min and a 1 µL injection. Mobile phase A consisted of water/acetonitrile (60/40) with 10 mM ammonium formate, while mobile phase B was 2-propanol/acetonitrile (90/10) and 10 mM ammonium formate. The column was equilibrated at 10% mobile phase B before a linear ramp to 50% at 2 min and a further increase to 100% mobile phase B at 8 min. This was held for 0.5 min before a return to 10% mobile phase B at 9 min. The column was equilibrated for 1 min prior to the next injection. The first 1 min of column flow was diverted to waste before being directed into the electrospray source (ES 5500 V) of a SCIEX QTRAP 6500 triple quadrupole tandem mass spectrometer in positive ion mode. Source conditions included an ion source temperature of 250 °C; curtain gas, 25 units; collision gas, medium; nebulizer gas 1, 20 units and auxiliary gas 2, 40 units. Individual species of Gb3 were quantified by multiple reaction monitoring (MRM) with concentrations calculated by comparing the area of the analyte to that of the IS. Total Gb3 was calculated by the sum of all isoforms. LysoGb3 from patient plasma samples was performed by Centogene (Rostock, Germany).

Additionally, α-Gal A activity was assessed using the Alpha Galactosidase Activity Assay Kit (Abcam), following the manufacturer's instructions with variations. Namely, the cells were lysed using six rounds of freezing and thawing in an ethanol, dry ice slurry, and the assay reaction mix included 100 mM N-acetylgalactosamine (Merck) to inhibit any α-Gal B activity. Cell protein concentration was measured using a micro bicinchoninic acid (BCA) assay kit (Thermo Fisher Scientific) and activity calculated in pmol/mg protein/hour.

Proteomic Analysis and Enrichment Studies

<u>Proteomic sample preparation</u>

Proteomic analysis was performed with liquid chromatography and tandem mass spectrometry (LC-MS/MS) on iPSC-derived podocytes using triplicate pellets via label-free quantification (LFQ). Samples were solubilised in 5 % SDS 100 mM Tris-HCl with heating at 95°C for 10 minutes to denature enzymes, DNA was sheared using probe sonication and subsequently samples were clarified at 13,000 rcf for 5 minutes. Protein quantification for sample normalization was performed utilizing a Pierce™ BCA Protein Assay Kit (Thermo Fisher Scientific). Further sample processing was performed using S-Traps ([5]; Protifi) utilizing Trypsin (Promega) at a 1:50 ratio of enzyme to protein. The resultant

peptides were purified by solid phase extraction utilzing stage-tips with polystyrene-divinylbenzene reversed-phase sulfonate resin (SDB-RPS; Merck).

Mass spectrometric analysis

Peptide mass amounts of 1 µg were injected for each sample onto LC-MS/MS system. Liquid chromatographic separation was performed on a Dionex UltiMate 3000 with an Acclaim PepMap RSLC analytical column (75 µm x 50 cm, nanoViper, C18, 2 µm, 100Å; Thermo Fisher Scientific) and an Acclaim PepMap 100 trap column (100 µm x 2 cm, nanoViper, C18, 5 µm, 100Å; Thermo Fisher Scientific), peptides were separated on in-house optimized gradients with increasing concentrations of 80% acetonitrile / 0.1 % formic acid at a flow rate of 250 nL/min for 120 minutes of linear separation. Nano-electrospray ionization of 2.0 kv was used into the Orbitrap Eclipse Tribrid mass spectrometer (Thermo Fisher Scientific) which operated in data-dependent analysis mode, and applied gas phase separation utilizing high field asymmetric waveform ion mobility spectrometry (FAIMS). The acquisition utilized two FAIMS compensation voltages (-45, -75) operated under default standard resolution with an ion transfer tube temperature of 300°C and a carrier gas flow rate of 4.6 L/min. Precursor ion scans were performed at a 60,000 resolution from 350 - 1,200 m/z, an RF lens of 40%, AGC target of 250% and ion injection time set to auto. Fragmentation scans were performed in the orbitrap at a resolution of 15,000 with normalized higher collision energy set to 28. Dynamic exclusion was applied for 45 seconds across all compensation voltages with only one charge state per precursor selected for fragmentation.

Data processing

The raw data files were analyzed with the Fragpipe software suite 17.1; (MSFragger version 3.4, Philosopher version 4.1.1). The label-free quantification match-between-runs (LFQ-MBR) workflow was applied with standard parameters, including two missed cleavages variable modifications of oxidised methionine and employing IonQuant and the MaxLFQ method of protein abundance calculations [9]. Searches were performed against the SwissProt human proteome (accessed June 2020) with common contaminants and indexed retention time (iRT) peptides (Biognosys).

Proteomic data analysis

Proteomic data analysis used the LFQ-Analyst platform. Briefly, differential protein abundances were determined using the *limma* package in Bioconductor with protein-wise linear modeling and empirical Bayes analysis, cut-offs were set at an adjusted p-value <0.05 and log2 fold-change greater than one. False discovery rate (FDR) correction was performed using Benjamini-Hochberg method. Enrichment analysis was performed using Gene Ontology (GO) and Kyoto Encyclopedia of Genes and Genomes (KEGG) databases to provide broader biological context. GO enrichment utilised the LFQ-Analyst Platform which presented enriched terms in bar charts for Cellular Component, Molecular Function, and Biological Process. KEGG enrichment was conducted using the ShinyGO platform, filtering

proteins based on p-value < 0.05, and presenting results in line charts for the 20 most enriched pathways based on the FDR and fold change.

A Venn diagram was generated to depict the overlap and uniqueness of significantly differentially expressed proteins between the control, $GLA^{c.851T>C}$, and $GLA^{c.1193\_1196del}$ iPSC-derived podocytes. For each sample group, the percentage of proteins displayed reflects both unique proteins and those shared with other groups. Percentages were calculated based on the number of significantly dysregulated proteins within each group, inclusive of shared and unique entries.
